# Karyotype variability in tropical maize sister inbred lines and hybrids compared with KYS standard line

**DOI:** 10.3389/fpls.2014.00544

**Published:** 2014-10-13

**Authors:** Mateus Mondin, Janay A. Santos-Serejo, Mônica R. Bertäo, Prianda Laborda, Daniel Pizzaia, Margarida L. R. Aguiar-Perecin

**Affiliations:** ^1^Department of Genetics, Luiz de Queiroz College of Agriculture, University of SãoPaulo, Piracicaba, Brazil; ^2^Embrapa Cassava and Fruits, Brazilian Agricultural Research CorporationCruz das Almas, Brazil; ^3^Department of Biological Sciences, Faculty of Sciences and Letters, São Paulo State UniversityAssis, Brazil; ^4^Center for Molecular Biology and Genetic Engineering, State University of CampinasCampinas, Brazil; ^5^Herminio Ometto University Center, Herminio Ometto FoundationAraras, Brazil

**Keywords:** maize, chromosomes, satellite DNA, centromere, heterochromatic knobs

## Abstract

Maize karyotype variability has been extensively investigated. The identification of maize somatic and pachytene chromosomes has improved with the development of fluorescence *in situ* hybridization (FISH) using tandemly repeated DNA sequences as probes. We identified the somatic chromosomes of sister inbred lines that were derived from a tropical flint maize population (Jac Duro [JD]), and hybrids between them, using FISH probes for the 180-bp knob repeat, centromeric satellite (CentC), centromeric satellite 4 (Cent4), subtelomeric clone 4-12-1, 5S ribosomal DNA and nucleolus organizing region DNA sequences. The observations were integrated with data based on C-banded mitotic metaphases and conventional analysis of pachytene chromosomes. Heterochromatic knobs visible at pachynema were coincident with C-bands and 180-bp FISH signals on somatic chromosomes, and most of them were large. Variation in the presence of some knobs was observed among lines. Small 180-bp knob signals were invariant on the short arms of chromosomes 1, 6, and 9. The subtelomeric 4-12-1 signal was also invariant and useful for identifying some chromosomes. The centromere location of chromosomes 2 and 4 differed from previous reports on standard maize lines. Somatic chromosomes of a JD line and the commonly used KYS line were compared by FISH in a hybrid of these lines. The pairing behavior of chromosomes 2 and 4 at pachytene stage in this hybrid was investigated using FISH with chromosome-specific probes. The homologues were fully synapsed, including the 5S rDNA and CentC sites on chromosome 2, and Cent4 and subtelomeric 4-12-1 sites on chromosome 4. This suggests that homologous chromosomes could pair through differential degrees of chromatin packaging in homologous arms differing in size. The results contribute to current knowledge of maize global diversity and also raise questions concerning the meiotic pairing of homologous chromosomes possibly differing in their amounts of repetitive DNA.

## INTRODUCTION

The identification of chromosomal features and the characterization of the maize genome structure have progressed extensively (Anderson et al., 2004; Kato et al., 2004; Schnable et al., 2009; Figueroa and Bass, 2012; Ghaffari et al., 2013), since the development of procedures for identifying maize meiotic chromosomes in the 20th century (McClintock, 1930; Longley, 1939; Rhoades, 1950). Karyotype analysis based on the observations of pachytene stage chromosomes obtained from pollen mother cells has contributed to important achievements in maize genetics (Creighton and McClintock, 1931; McClintock, 1950; Carlson, 1988; Coe, 1994). The construction of the earliest detailed meiotic cytogenetic maps (Neuffer et al., 1997) involved the location of heterochromatic regions, such as cytologically observable knobs and centromeric heterochromatin, in addition to the identification of chromosomes by relative length and arm ratio.

The size and number of knobs are variable and they may be present in each of the 10 chromosomes of the complement at fixed locations on the chromosome arms in modern maize and its relatives, including species of *Zea* (teosintes) and *Tripsacum* (McClintock et al., 1981). Knobs have been used extensively as chromosomal markers (e.g., Ghaffari et al., 2013) and to study race relationships and the phylogenetic history of *Zea mays* (McClintock, 1978; McClintock et al., 1981).

Meiotic chromosome analysis continues to be an important tool for maize cytogenetics; on the other hand, procedures for the identification of maize mitotic chromosomes in root tip spreads have been developed. The examination of C-banded somatic metaphases has allowed the detection of bands corresponding with knobs visualized on meiotic chromosomes (Aguiar-Perecin and Vosa, 1985) and proven to be useful for the detection of homozygous and heterozygous knobs in many individuals in a population. However, the unequivocal identification of somatic chromosomes may be difficult due to the degree of chromatin condensation and the presence of large knobs that alters the sizes of the chromosome arms. Thus, this type of procedure must be supplemented with an analysis of pachytene stage chromosomes, in which details of chromosomal structure can be visualized. The examination of C-banded metaphases has been useful in some studies, such as those involving the detection of changes in chromosome 7 resulting from breakage or amplification events at knob sites in cells of callus cultures (Fluminhan et al., 1996; Aguiar-Perecin et al., 2000). Also, C-banding was used to determine the number of knobs in a work aiming to evaluate the DNA content in maize populations (Rayburn et al., 1985).

Chromosome identification has improved with the mapping of repetitive DNA sequences by fluorescence *in situ* hybridization (FISH) on maize meiotic and mitotic chromosomes. Many groups of eukaryotes, such as mammals and higher plants, have genomes that are composed mainly of repetitive DNA that can be divided into two categories, including tandem repeat arrays (satellite DNA) and transposable elements (TEs; revised by Heslop-Harrison and Schwarzacher, 2011). Satellite DNA accumulates at specific chromosomal regions, particularly at heterochromatic sites, such as maize knobs, that are composed primarily of two tandemly repeated sequences, the 180-bp knob repeat or the 350-bp TR-1 element or a mixture of both (Ananiev et al., 1998a; Kato et al., 2004; Albert et al., 2010; Ghaffari et al., 2013). Maize centromeres are composed of arrays of the CentC satellite (monomer length ∼156 nt; Ananiev et al., 1998b) and interspersed centromeric–specific retrotransposons (CRM; Zhong et al., 2002; Nagaki et al., 2003; Jin et al., 2004). Other repetitive DNA sequences, such as nucleolus organizing region (NOR) DNA, 5S ribosomal DNA (5S rDNA), centromeric satellite 4 (Cent4), subtelomeric sequences and microsatellites have been mapped to maize chromosomes and are valuable landmarks for chromosome identification (Chen et al., 2000; Page et al., 2001; Kato et al., 2004). FISH analyses using probes of some of these satellite DNAs have allowed the study of karyotype diversity in maize inbred lines commonly used in cytogenetic and genetic studies, including e.g., B73, KYS, and Mo17 among others and in lines of a nested association mapping (NAM) population (Albert et al., 2010). Other reports on the FISH mapping of repetitive DNA and genes have provided valuable information on the evolution of maize chromosomal features (Lamb and Birchler, 2006; Lamb et al., 2007b; Danilova and Birchler, 2008).

In this study, we analyzed the somatic karyotypes of tropical maize lines and hybrids using FISH mapping of tandemly repeated DNA sequences. We investigated the karyotypes of S6 sister inbred lines that were derived from a flint maize population [Jac Duro (JD)]. Our objective was to integrate a previous cytogenetic analysis (unpublished) based on classical techniques with the FISH data. Observations using C-banding and FISH mapping of somatic chromosomes and a conventional analysis of pachytene chromosomes were compared. The FISH procedure employed was useful for chromosome identification and to detect that the centromere position of chromosomes 2 and 4 differed from the pattern reported for standard lines (Neuffer et al., 1997). Thus, the somatic karyotypes of one JD line and the standard line KYS were compared by the FISH mapping of satellite DNA sequences using a hybrid between these lines. The pairing behavior at pachytene stage of chromosomes 2 and 4 was also investigated in this hybrid. Because most of maize cytogenetic and genome structure reports have been based on temperate maize inbreds, the objective of our work was also to contribute to the knowledge of maize global diversity and evolution.

## MATERIALS AND METHODS

### PLANT MATERIAL

The seeds of sister lines were obtained in our laboratory by sibling crosses of S6 progenies that were developed from one S2 progeny derived from a sample of the maize flint JD population (Sementes Agroceres, Brazil). The JD population was composed of Cateto (Cateto São Simão and Cateto Minas Gerais II) and Cuba varieties (personal communication, Dr. Urbano C. Ribeiral, Agroceres). A previous examination of somatic C-banded metaphases of S2 progeny plants showed that C-bands (knobs) localized on the long arms of chromosomes 3 and 5 (3L and 5L) and on the short arms of chromosomes 7 and 9 (7S and 9S) were segregating and the long arms of chromosomes 6, 7, and 8 (6L, 7L, and 8L) were homozygous for C-bands in all of the plants that were examined (Decico, 1991; **Figure S1** in Supplementary Material). The lines selected for the present work belonged to the JD 1-3 and JD 4-4 families (**Table 1**; **Figure S1**), which were previously used in a survey of their embryogenic response in callus cultures (Fluminhan and Aguiar-Perecin, 1998). In addition, the karyotypes of hybrids between JD lines and of a hybrid between the 441311 line and the temperate KYS line were analyzed. The KYS seeds were provided by the Maize Genetics Cooperation Center (USA).

**Table 1:** JD 1-3 and JD 4-4 line families analyzed and their knob compositions visualized in pachytene chromosomes.

Lines	Knob composition								
	K3L	K5L	K6L2	K6L3	K7S	K7L	K8L1	K8L2	K9S
**JD 1-3 family**
132331	00	00	++	++	++	++	++	++	++
133425	00	00	++	++	++	++	++	++	00
**JD 4-4 family**
441123	++	++	++	++	++	++	++	++	++
441311	++	++	++	++	++	++	++	++	++
442612	00	++	++	++	00	++	++	++	++
444331	00	++	++	++	00	++	++	++	00

*K, knob; numbers refer to chromosomes; L, long arm; S, short arm; L1, L2, and L3 refer to different knob positions on the same chromosome arm; +, presence of knob; 0, absence of knob.*

### PREPARATION OF CHROMOSOME SPREADS

For mitotic analyses, kernels were germinated at 28°C for 2–3 days, and excised roots were pretreated with a solution containing 300 mg/L 8-hydroxiquinoline and 1.25 mg/L cycloheximide for 2.5 h at 28^o^C, fixed in 3:1 ethanol:acetic acid and stored at -20^o^C. Metaphase spreads were prepared by treating the roots with 45% acetic acid (for the C–banding protocol) or with 60% acetic acid (for the FISH protocol) for 5 min, after which the root tips were dissected, and the meristematic cells were squashed. The coverslips were removed in liquid nitrogen, air dried and stored at –20°C until use.

The Giemsa C-banding was carried out as previously described (Bertão and Aguiar-Perecin, 2002) to reveal features of the somatic chromosomes of the 441123 and 444331 lines and the 441123 × 444331 and 132331 × 134425 hybrids.

For meiotic chromosome preparations, immature tassels were fixed in 3:1 ethanol:acetic acid and kept at -20°C. For the conventional observation of pachytene chromosomes the anthers were dissected in 1% propionic carmine (prepared in 45% propionic acid) and the microsporocytes were squashed. The 441311, 444331, and KYS lines and the 441123 × 444331 and 441311 × KYS hybrids were used in this analysis. In FISH experiments, the anthers with meiotic cells at the pachytene stage from the 441131 × KYS hybrid were selected using light microscopy, by examining the cells of one anther stained with 0.5% acetocarmine. The remaining anthers of each spikelet were washed in water and digested in pectinase (Calbiochem 515883, Germany; final concentration of 14.7 units/mL) and cellulase (Serva 16420, Germany; final concentration of 9.2 units/mL) for 20 min at 37°C (two anthers per tube with 100 μL of enzyme solution). After the digestion, they were washed in cold distilled water and in 60% acetic acid for 2 min and squashed. The coverslips were then removed in liquid nitrogen and the slides were air-dried.

### FISH PROCEDURE

Probes for the primary knob 180-bp repeat (Peacock et al., 1981) and for the centromeric repeat CentC (Ananiev et al., 1998b) were used. Chromosome-specific probes (Kato et al., 2004) were used to identify chromosome 2 (5S rDNA), chromosome 4 (Cent4), chromosome 6 (9.1-kb repeating unit of the NOR DNA) and other specific chromosomes (subtelomeric 4-12-1 clone). The Cent4 and subtelomeric probes were provided by Dr. J. A. Birchler (Missouri University, USA), and the 180-bp and NOR rDNA probes by Dr. R. L. Phillips (Minnesota University, USA). The 5S rDNA probe was a fragment of about 450-bp (gene and spacer) that was amplified by PCR using total genomic DNA extracted from the leaves of seedlings from the 441311 line. This PCR procedure was carried out as described by Mondin et al. (2007). The CentC probe was a sequence consisting of two CentC consensus repeats (Ananiev et al., 1998b), including one of 154-bp and the other of 139-bp (GenBank accession no. KJ466900; **Figure S2** in Supplementary Material). This dimer was obtained from genomic DNA that was isolated from the AL-739 maize line (Germplasm Bank of IAC, Campinas, SP, Brazil), employing the CTAB protocol (Hoisington et al., 1994). The sequence was identified in a library obtained from PCR products using the primers F-5′-GGTTCCGGTGGCAAAAACTCGT-3′ and R-5′ ATTTCTTCGTTTTTCACAACGAACATG-3′ consensus for a CentC region. The amplifications were carried out using the touchdown PCR method with temperature varying from 65 to 55°C, and the PCR products were cloned into TA Cloning vector (Invitrogen, USA). Selected clones were sequenced using the ABI PRISM *BigDye Terminator Cycle Sequencing* Kit (Applied Biosystems, USA) in the ABI 377 sequencer. Clone H9, in which the CentC dimer was identified, was used to obtain the FISH probe. Then, the amplification of this sequence was carried out using the primers F-5′-GTGTGGAATTGTGAGCGGATAAC-3′ and R-5′-TTGTAAAACGACGGCCAGTGAAT-3′ (complementary to the vector sequence).

The Cent4, CentC, NOR rDNA and subtelomeric probes were labeled with biotin-14-dATP by nick translation (Bionick Labelling System, Invitrogen, USA) and detected with mouse anti-biotin followed by TRITC-conjugated rabbit anti-mouse (red) and TRITC-conjugated swine anti-rabbit antibodies (DAKO, Denmark), with the exception of the KYS × 441311 hybrid somatic karyotype, for which the CentC probe was detected with FITC-conjugated antibodies (green). The NOR rDNA was detected using a mixture of 50% rabbit anti-mouse FITC and 50% anti-mouse TRITC, resulting in a yellow signal. The knob180-bp and 5S rDNA sequences were labeled with digoxigenin 11-dUTP (Roche, Germany) by random priming and detected with FITC- or rhodamine-conjugated sheep anti-digoxigenin (Roche; red).

The FISH procedure was performed as previously described (Mondin et al., 2007) with minor modifications. Each cell preparation was carried out using 20 μL of the probe mixture containing two to three probes (10–20 ng/μL of each probe). The probes were denatured by heating at 96°C for 10 min, cooled in ice, and then dropped onto slide preparations onto which coverlips were applicated. The preparations were denatured in a thermocycler at 93°C for 10 min. The hybridization was performed at 37°C for 16–20 h. Post-hybridization steps followed the protocol previously described (Mondin et al., 2007). The slides were counterstained with DAPI (1 μg/mL) and mounted using Vectashield (Vector, USA).

### IMAGE CAPTURE, PROCESSING AND KARYOTYPE ANALYSIS

The C-banded metaphases and carmine stained pachytene chromosomes were photographed on Technical Pan Film (Kodak) using a Zeiss photomicroscope, with the exception of the pachytene chromosomes from the hybrid 441311 × KYS which were examined using a Zeiss Axiophot 2 microscope, and the images were acquired by a CCD camera and analyzed with the IKAROS software (MetaSystems, Germany). FISH images were observed under this microscope, with the appropriate filters and analyzed with the ISIS software (MetaSystems). All of the images were processed with Adobe Photoshop 6.0.

The chromosomes of somatic C-banded metaphases, with the same degree of condensation, from the 441123, 444331 lines and 441123 × 444331 hybrid were measured, and their relative lengths (expressed as percent of chromosome 10, as reported by Aguiar-Perecin and Vosa, 1985) and arm ratios were estimated. An ideogram that was based on the data from 15 metaphases was outlined. Pachytene chromosomes of the hybrid 441123 × 444331 were measured to estimate their arm ratios. The chromosome relative lengths were not scored because it was difficult to distinguish all of the chromosomes in the same pachytene cell, due to the fusion of large knobs. About 10 individual chromosomes were measured.

The arm ratios of chromosomes 2, 4, and 5, at pachytene stage, from 441311, 444331, and KYS lines and hybrid 441311 × KYS were estimated and tested through an analysis of variance and mean values were compared by their confidence intervals (Faraway, 2004). Images of about 20 cells from 2 to 3 plants of each material were used for the chromosome arm measurements.

## RESULTS

### CHARACTERIZATION OF MITOTIC AND MEIOTIC CHROMOSOMES

**FIGURE 1:**
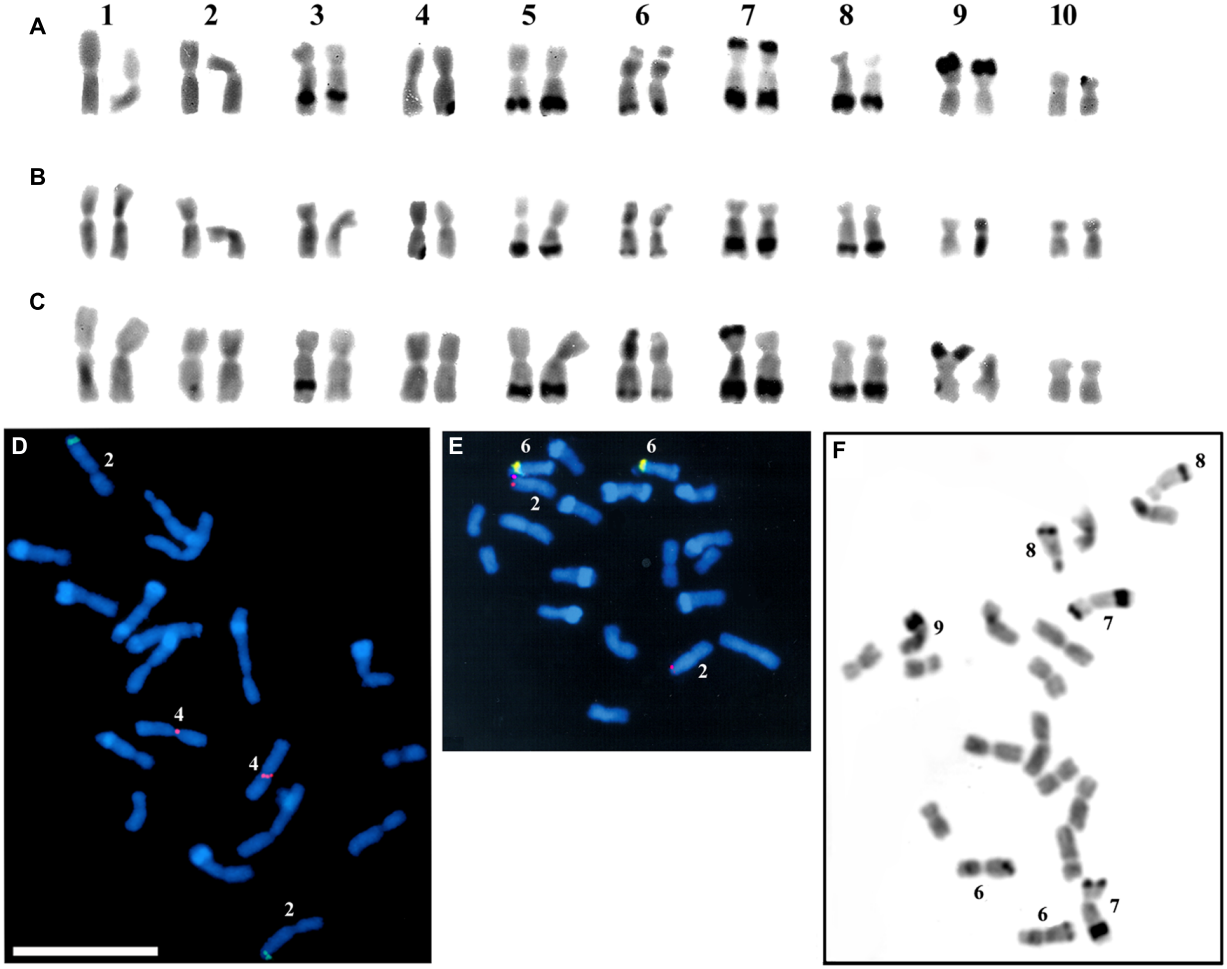
**Somatic chromosomes of JD lines and hybrids.** C-banded karyograms from lines 441123 **(A)**, 444331 **(B),** and the 441123 × 444331 hybrid **(C)**; metaphase of the 441123 × 442612 hybrid showing 5S rDNA (green) and Cent4 (red) FISH signals **(D)**; metaphase of the 441123 × 444331 hybrid with 5S DNA (red) and NOR rDNA (yellow) FISH signals **(E)**; C-banded metaphase of the 132331 × 133425 hybrid **(F)**, in which the knobbed chromosomes are identified. Scale bar = 10 μm.

The identification of the C-banded somatic chromosomes was based on their relative lengths and arm ratios (**Table 2**), and on the knob (C-band) positions. **Figure 1** shows the C-banded karyograms of the 441123 and 444331 lines and their hybrid. In the 441123 line (**Figure 1A**), knobs were observed on chromosomes 3L, 5L, 6L 7SL, 8L, and 9S. In the 444331 line, the knobs on 3L, 7S, and 9S were not present (**Figure 1B**), and chromosome 7 could be distinguished from chromosome 8 by its larger knob (K7L). In the 441123 × 444331 hybrid (**Figure 1C**), it can be visualized that in the heterozygous pairs 3, 7, and 9, the presence of the large knob altered the chromosome sizes (**Table 2**). Chromosomes 2 and 4 were recognized in the C-banded metaphases according to FISH data using the chromosome-specific 5S rDNA probe for chromosome 2 (Mascia et al., 1981) and Cent4 for chromosome 4 (Page et al., 2001), as seen in the metaphase from a 441123 × 442612 hybrid (**Figure 1D**). The position of the chromosome 2 centromere was submedian in comparison with that of chromosome 4. It is interesting to note that the FISH procedure enhances the visualization of the knob regions, which can be seen as DAPI bands. The knob composition of the 442612 line is shown in **Table 1**. Chromosome 6 was identified by the NOR rDNA signal (**Figure 1E**), and by the secondary constriction and satellite on 6S which were visualized in the C-banding and FISH spreads (**Figures 1** and **4**). In the 132331 and 133425 lines, knobs on 3L and 5L were absent as can be observed in the 132331 × 133425 hybrid, homozygous for knobs at 6L, 7SL, and 8L, and heterozygous for K9S (**Figure 1F**), which was only observed in 132331 line (**Table 1**). The values of relative lengths and arm ratios of the somatic chromosomes without knobs (**Table 2**) were consistent with previous data describing wild maize (Aguiar-Perecin and Vosa, 1985), except for the arm ratios of chromosomes 2 and 4, higher in chromosome 2 (about 1.44) compared with chromosome 4 (about 1.30) in JD lines. The knobless chromosome 5 was analyzed in spreads of the 132331 × 133425 hybrid.

**Table 2:** Relative lengths and arm ratios of somatic chromosomes and arm ratios of pachytene chromosomes in JD lines. K6L2/L3 and K8L1/L2 are detected as a single band in mitotic chromosomes.

Chromosome features	Chromosome rank													
	1	2	3		4	5		6	7		8	9		10
			KL	*		KL	*	KL2/L3	KS/KL	KL	KL1/L2	KS	*	
**Metaphase**
RL	178.1	149.3	155.6	146.9	143.0	158.9	135.9	120.3	159.4	137.5	131.3	137.5	106.7	100
AR	1.24	1.44	1.97	1.85	1.30	1.35	1.02	1.85	1.83	2.83	3.0	0.91	1.64	1.81
**Pachytene**
AR	1.28	1.71	2.59		1.44	1.13		3.82	2.69		3.82	1.59		2.68

RL, relative length (expressed as percent of chromosome 10 length); AR, arm ratio; KL, knob on the long arm; KS, knob on the short arm; KL2/L3, knob positions on chromosome 6; KL1/L2, knob positions on chromosome 8; *without knob.

**FIGURE 2:**
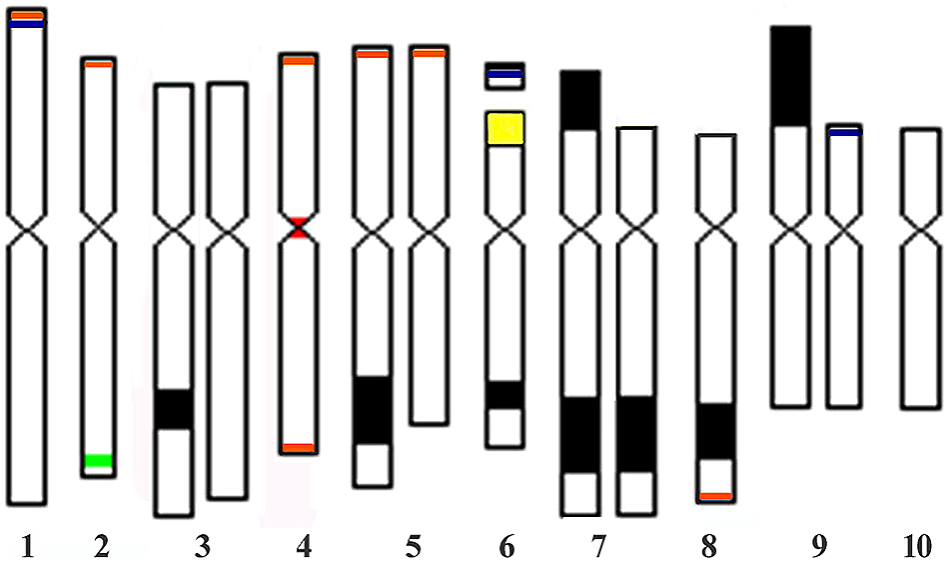
**Ideogram representative of the somatic chromosomes of JD lines showing knobs on chromosomes 3, 5, 6, 7, 8, and 9, detected by C-banding and FISH probed with the knob 180-bp repeat (black), small 180-bp repeat signals (blue), subtelomeric 4-12-1 clone (orange), Cent4 (red), 5S rDNA (green), and NOR rDNA (yellow).** Knobless homologues of chromosomes 3, 5, 7, (without K7S), and 9 are displayed.

From these observations on the C-banded somatic chromosomes, we delineated an ideogram showing the chromosomes with and without knobs (**Figure 2**).

**FIGURE 3:**
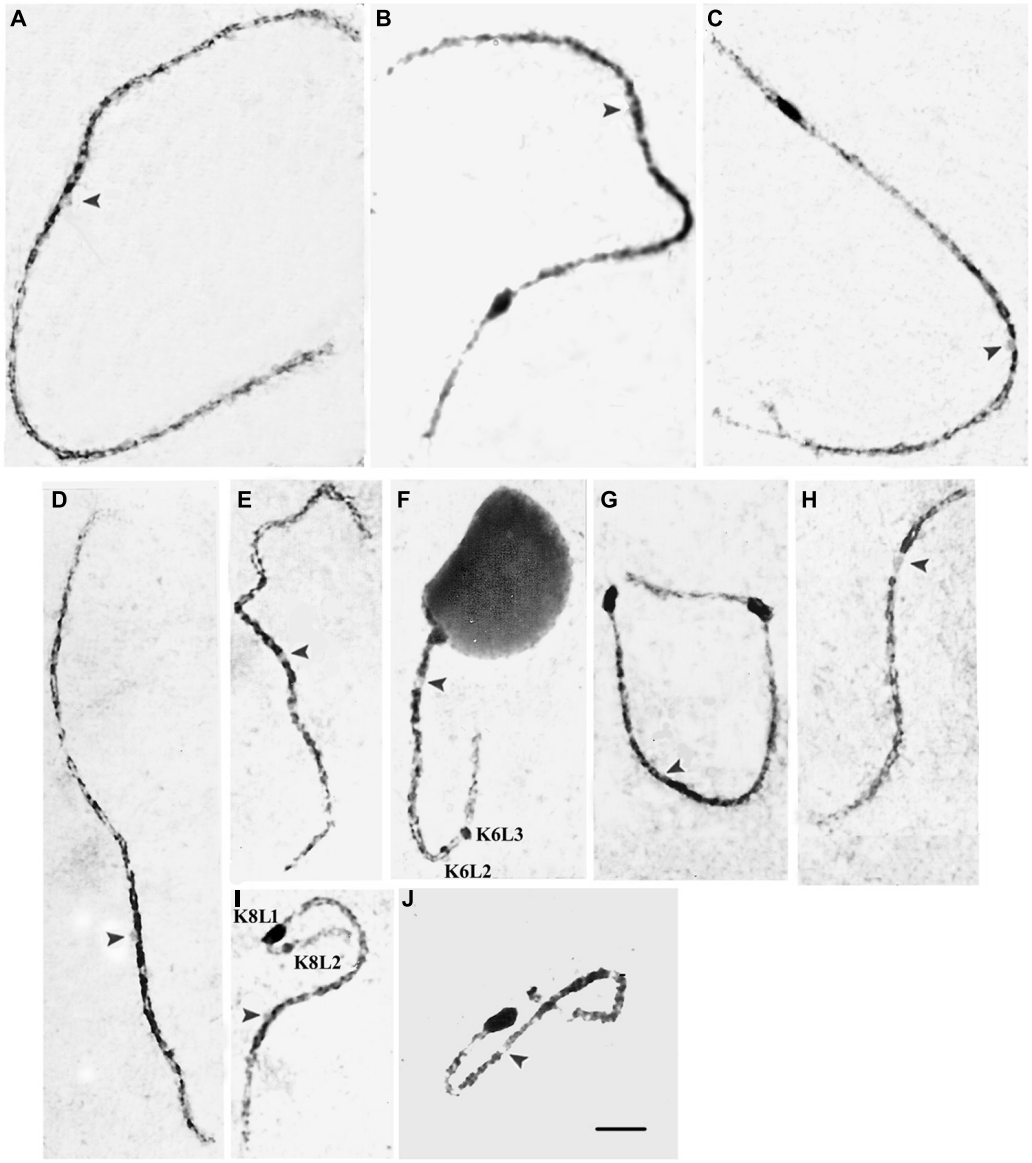
**Carmine-stained pachytene chromosomes of the 441123 × 444331 hybrid showing cytologically visible knobs on chromosomes 3**(B)**, 5**(C),** 6**(F),** 7**(G),** 8**(I),** and 9**(J)**.** Chromosomes 1**(A)**, 2**(D)**, 4**(E),** and 10**(H)** do not possess visible knobs. Note the presence of two knobs on 6L and 8L. Arrowheads indicate centromeres. Scale bar = 5 μm.

The carmine-stained pachytene chromosomes of the 441123 × 444331 hybrid, identified by their sizes, arm ratios and knob positions are depicted in **Figure 3**. The cytologically visible knobs corresponding with the C-bands detected in mitotic chromosomes on 3L, 5L, 7SL, 8L, and 9S were large (**Figures 3B,C,G,I,J**). There were two small knobs on the long arm of chromosome 6 (6L2 and 6L3; **Figure 3F**), which appeared as a thin unique band on the somatic chromosomes. L2 and L3 refer to distal positions, as reported in the classical literature (McClintock et al., 1981). Two knobs were observed on the long arm of chromosome 8: 8L1 (large) and 8L2 (small; **Figure 3I**), which also appeared as a unique band in somatic metaphases. Chromosomes 1, 2, 4, and 10 (**Figures 3A,D,E,H**) had no distinguishable knobs. The arm ratio values that were estimated for the pachytene chromosomes from the 441123 × 444331 hybrid (**Table 2**) are in agreement with reports on the characterization of maize pachytene chromosomes (Rhoades, 1950; McClintock et al., 1981; Dempsey, 1994), with the exception of the chromosomes 2 and 4, which possessed arm ratios of 1.71 and 1.44, respectively.

**FIGURE 4:**
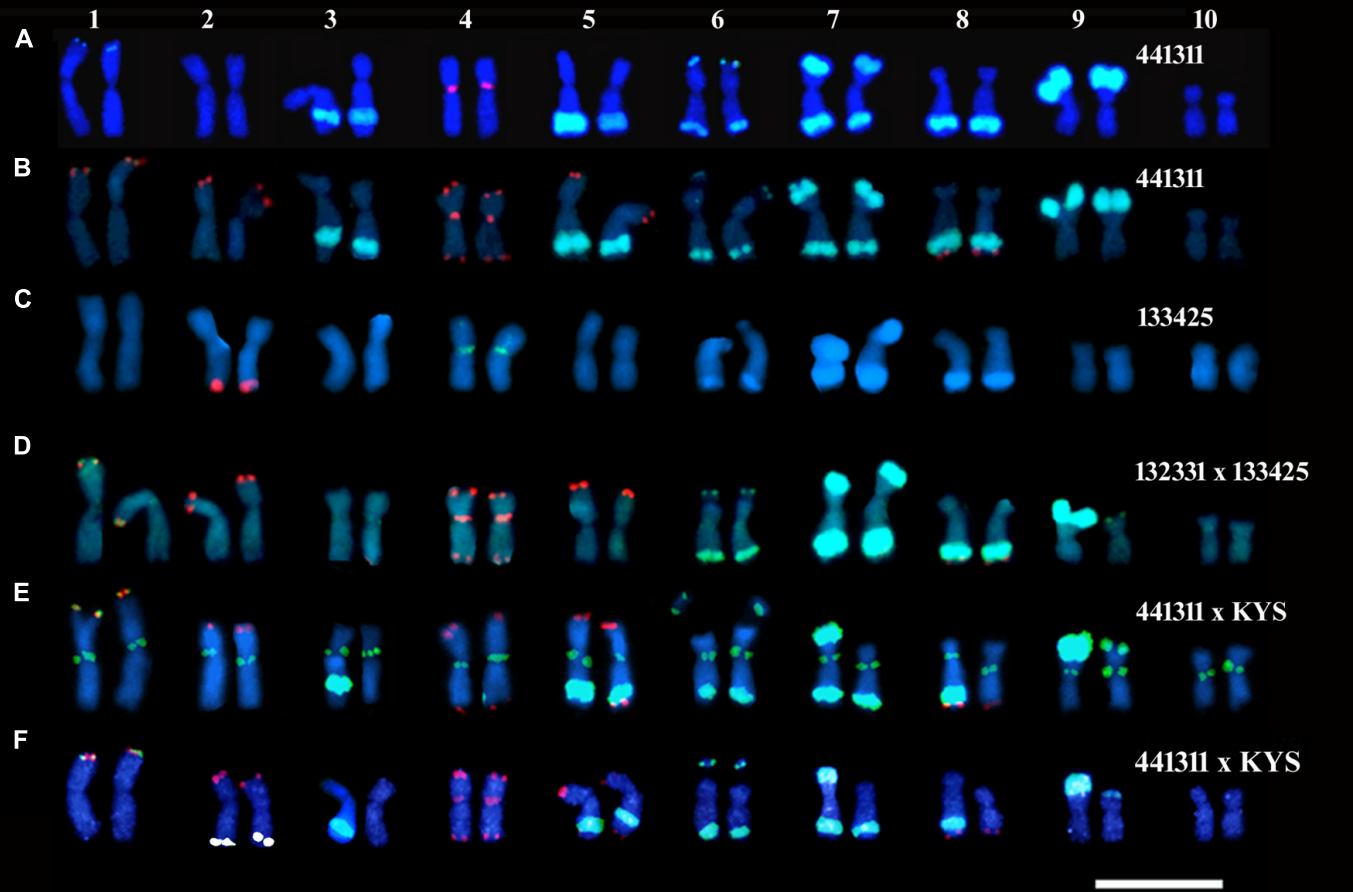
**Somatic karyotypes of JD lines **(A–C)** and hybrids **(D–F**), labeled by FISH with probes for the knob 180-bp repeat (**A**,**B**,**D–F**, green), Cent4 (**A**,**B**,**D,F**, red and **C**, green), subtelomeric 4-12-1 (**B**,**D**–**F**, red), 5S rDNA (**C**, red and **F**, pseudo-colored white), CentC (**E**, green).** Note that the large knobs can be detected as DAPI bands in **(C)** and that the knobless chromosome 9 from the parent 133425, in hybrid 132331 × 133425, has a small 180-bp signal on 9S **(D)**. Chromosomes 2, 3, 5, 7, 8, and 9 from the 441311 parent (placed on the left) can be recognized in the 441311 × KYS hybrid **(E,F)**. The chromosomes were counterstained with DAPI. Scale bar = 10 μm.

Fluorescence *in situ* hybridization signal locations of the satellite DNA sequences were determined in the 441311 and 133425 lines and the 133425 × 132331 and 441311 × KYS hybrids. The 441311 line was closely related to 441123 and had the same knob composition (**Table 1**). KYS is a maize line commonly used in cytogenetic research, and its chromosomes have been well characterized for size, arm ratio and the sequence distributions, which were investigated here (Chen et al., 2000; Anderson et al., 2003; Kato et al., 2004). Separate hybridizations were performed to permit a reliable visualization of overlapping signals. The locations of signals on the chromosomes from the 441311 line were as follows: the 180-bp knob repeat was detected on 1S, 3L, 5L, 6SL, 7SL, 8L, and 9S; the Cent4 satellite was visualized at the primary constriction of chromosome 4 (**Figures 4A,B**). The signals of the 180-bp sequence on 1S and 6S were very small and the others corresponded in sizes and positions to the large knobs that were observed in the 441123 sister line (**Figure 1A**). The subtelomeric 4-12-1 sequence was detected on 1S, 2S, 4SL, 5S, and 8L (**Figure 4B**). The 5S rDNA signal that was observed on the submetacentric chromosome 2 is displayed in **Figure 1D** in the 441311 × 442612 hybrid, as mentioned above. In the 133425 line, chromosome 2 with the 5S rDNA signal (red) also showed the centromere located at a submedian position in comparison with metacentric chromosome 4 labeled with Cent4 signal (green, **Figure 4C**). 180-bp knob signals corresponding with visible knobs were detected on 6L, 7SL, 8L and 9S in the 132331 × 133425 hybrid and small signals were also observed in 1S and 6S (**Figure 4D**). The signal of the large K9S, present in 132331 line, was observed in the hybrid 132331 × 133425 and the knobless chromosome 9 from the parent 133425 had a thin 180-bp signal on the tip of 9S (**Figure 4D**), not detected as a DAPI band in 133425 (**Figure 3C**). The lines of the family JD 1-3 did not possess a knob on 5L. The subtelomeric 4-12-1 signals were in the same positions as seen in the 441311 line. These sequence locations were included in the ideogram representing the somatic karyotype of JD lines (**Figure 2**).

The hybrid 441311 × KYS allowed for the comparison of chromosomes from both parents in the same FISH conditions and same degree of chromosome condensation (**Figures 4E,F**). The CentC signals (green, **Figure 4E**), detected by three antibodies, were strong in most of the chromosome pairs and did not discriminate specific chromosomes. The 4-12-1 subtelomeric sequence was on the same position observed for the JD lines, with exception of chromosome 5, on which it was present at S and L in the KYS homologue. The 5S rDNA (pseudo-colored write) and Cent4 signals (**Figure 4F**) were located on chromosomes 2 and 4 as described above for JD lines. The 180-bp knob sequence signals on the KYS chromosomes were at 1S, 5L, 6SL, 7L, and 9S as expected according to previous reports (Kato et al., 2004). The K5L, K7L, and K9S signals were smaller than the homologous signals from the 441311 parent. Most of the chromosomes from 441311 could be recognized in this hybrid, and were placed on the left side of each homologous pair.

From these observations, the markers that may be used to unambiguously identify the somatic chromosomes of the JD lines (**Figures 2** and **4**) are as follows.

Chromosome 1. Small 180-bp knob repeat and 4-12-1 subtelomeric FISH signals at the tip of the short arm.

Chromosome 2. 5S rDNA signal on 2L and 4-12-1 subtelomeric signal on 2S.

Chromosome 3. No special markers were detected and this chromosome can be distinguished by its arm ratio ∼1.85 in knobless homologues, or large knob almost on the center of the long arm.

Chromosome 4. Cent4 satellite signal at the centromere and 4-12-1 subtelomeric signals on SL.

Chromosome 5. 4-12-1 subtelomeric signal on 5S.

Chromosome 6. NOR-rDNA, secondary constriction and small 180-bp knob signal on the tip of the short arm.

Chromosome 7. Large knob on 7L in all of the JD lines.

Chromosome 8. 4-12-1 subtelomeric signal and large knob on 8L in all of JD lines.

Chromosome 9. Large knob on 9S or the very small 180-bp knob signal on the tip of the short arm in knobless homologues.

Chromosome 10. No special markers were detected, but it is distinguishable by its small size.

### BEHAVIOR OF THE CHROMOSOMES 2 AND 4 AT PACHYTENE STAGE IN THE 441311 × KYS HYBRID

**FIGURE 5:**
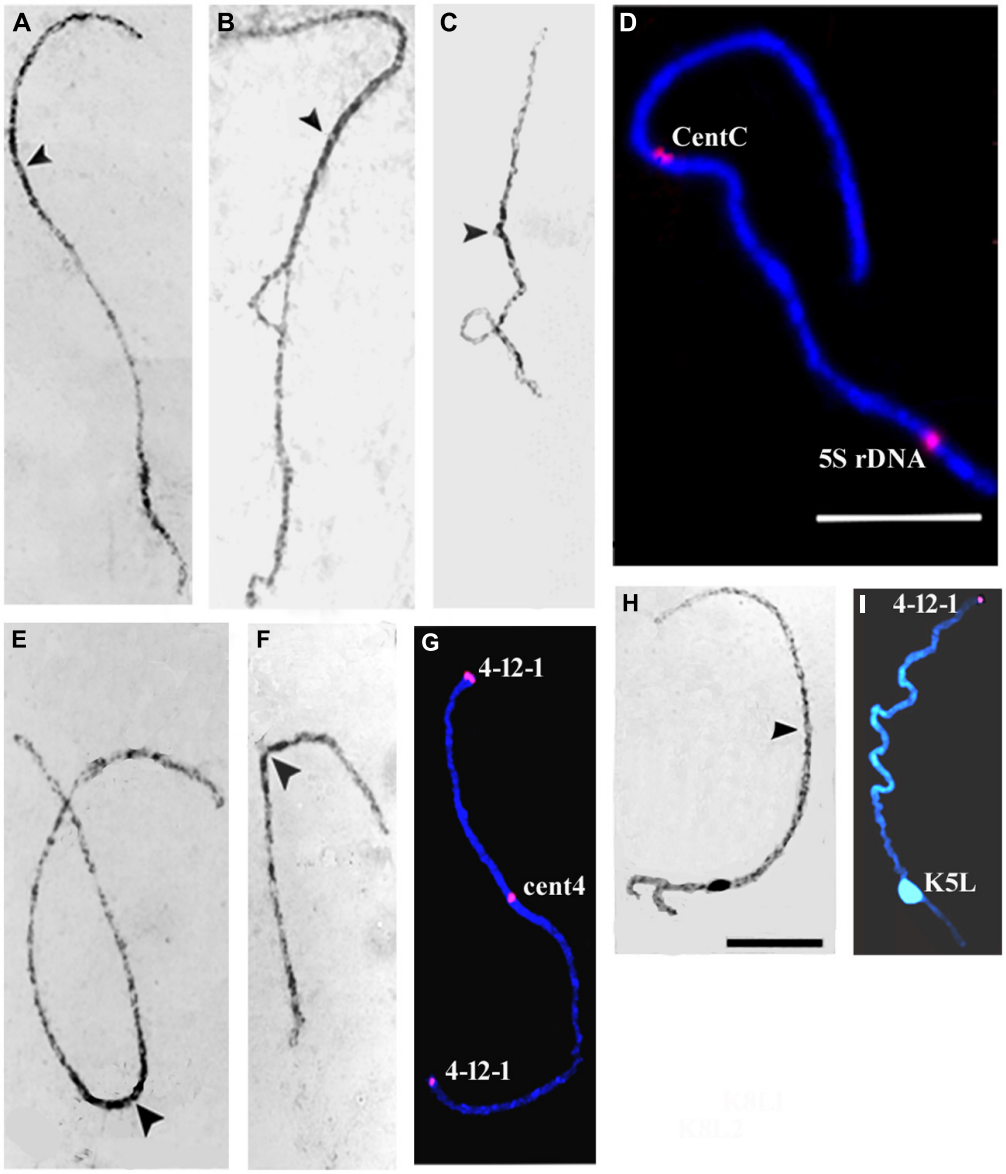
**Chromosomes 2 **(A–D)**, 4 **(E–G),** and 5 **(H,I)** at pachytene stage from the 441311 × KYS hybrid **(A**,**B**,**D**,**E**,**G,H)** and KYS **(C**,**F)** and 444331 **(I)** lines.** The homologues are completely synapsed in the hybrid, with exception of one cell in which a loop possibly resulting from a pairing failure occurred in a segment of the bivalent 2 **(B)**. The arrowheads indicate the centromeres in carmine-stained chromosomes **(A–C,E,F,H)**. FISH signals (red) of CentC and 5S rDNA probes on chromosome 2 **(D)**, Cent4 and subtelomeric 4-12-1 clone (red) on chromosome 4 **(G)**, and 4-12-1 on chromosome 5 **(I)** are displayed. Scale bars for carmine stained chromosomes and FISH images = 10 μm.

The pachytene chromosomes of the 441311 × KYS hybrid were examined to investigate the pairing behavior of chromosomes 2 and 4. Carmine-stained chromosomes 2 and 4 from the 441311 and KYS lines and the respective hybrid (**Figures 5A–C,E,F**) were analyzed to compare their arm ratios. Chromosomes of the 444331 line were also included in this analysis (**Table 3**). The arm ratios of the chromosome 2 of 441311, 444331 and 4411311 × KYS were very similar (about 1.70), while in KYS (**Figure 5C**) it was 1.36, corresponding to data in the literature (**Table 3**; see review of KYS data in Anderson et al., 2003). The homologous chromosomes of the bivalent 2 were completely synapsed in all of the cells that were examined (**Figure 5A**), and in only one microsporocyte, a loop was detected (**Figure 5B**), suggesting the occurrence of a pairing failure in a chromosomal segment. The chromosome 4 arm ratios in 441311, 444331 and 4411311 × KYS were not similar (about 1.37 in the lines and 1.47 in the hybrid). Therefore, the centromeric position of the bivalent 4 appeared to be more variable among cells in the hybrid, but the pairing between homologues was complete (**Figure 5E**). The chromosome 4 arm ratio of KYS (**Figure 5F**) estimated in the present study was 1.63, which is also consistent with data in the literature. Therefore, the data showed that in the hybrid, the arm ratio value of chromosome 2 was similar to the one of the JD lines, while the chromosome 4 arm ratio was significantly different from the JD lines and intermediate between the parents (**Table 3**).

**Table 3:** Arm ratios of chromosomes 2, 4, and 5 at pachytene stage from the 441311, 444331, and KYS lines and the 441311 × KYS hybrid compared with KYS data from various studies.

Materials	Chromosomes		
	2	4	5
441311	1.69 (1.63; 1.75) a	1.37 (1.33; 1.41) c	1.09 (1.00; 1.18) a
444331	1.70 (1.61; 1.79) a	1.36 (1.30; 1.41) c	1.12 (1.06; 1.19) a
KYS^∗^	1.36 (1.27; 1.44) b	1.63 (1.54; 1.71) a	1.04 (0.97; 1.11) a
441311 × KYS	1.69 (1.62; 1.77) a	1.47 (1.42; 1.52) b	1.09 (1.02; 1.16) a
Rhoades (1950)^#^	1.26	1.59	1.20
McClintock et al. (1981)^#^	1.20	1.57	1.16
Dempsey (1994)^#^	1.25	1.60	1.10

*^∗^Present study; ^#^KYS data from other studies. Chromosome arm ratio for each line and hybrid were tested through an analysis of variance. Values with the same letter are equal based on their confidence intervals in brackets.*

Fluorescence *in situ* hybridization using chromosome-specific probes to identify chromosomes 2 and 4 showed features of the chromosome pairing in the 441311 × KYS hybrid. The bivalent 2, labeled with the CentC and 5S rDNA probes (**Figure 5D**), and the bivalent 4 labeled with the Cent4 and 4-12-1 subtelomeric clone probes (**Figure 5G**), showed normal synapsis at the repetitive DNA sites analyzed. Additionally, it is interesting to note that the other chromosomes presented normal pairing in this hybrid and we included here observations on chromosome 5, as example. The pair 5, which possessed knobs of different sizes on 5L in the homologues, showed normal synapsis at this region (**Figure 5H**). As in KYS this knob is located at 5L, the analysis of this hybrid was also important to give additional evidence that the knob of the metacentric chromosome 5 is also located on 5L, in the JD lines. This can be seen in chromosome 5 of the 444331 line with the large knob on 5L and the 4-12-1 subtelomeric signal on 5S (**Figure 5I**). The arm ratios for the JD and KYS lines and the hybrid studied here were consistent with the median position of the centromere in chromosome 5 (**Table 3**).

## DISCUSSION

### POLYMORPHISM OF MAIZE CHROMOSOMES

The current FISH protocol using probes of repetitive DNA sequences efficiently characterized the somatic chromosomes of the inbreds and hybrids investigated. In a study on the embryogenic response in callus cultures from JD lines (Fluminhan and Aguiar-Perecin, 1998), and another on cytogenetic techniques (Bertão and Aguiar-Perecin, 2002), the true chromosomes 2 and 4 were identified as 4 and 5, based on their arm ratios, and the actual knobbed chromosome 5 was identified as chromosome 2. The problems with regard to the identification of chromosomes 2 and 4 were due to the submedian positioning of the centromere of chromosome 2 in comparison with that of chromosome 4, not expected according to the literature, as mentioned above. The mapping by FISH of the 5S rDNA and Cent4 chromosome-specific sequences was important for the reliable identification of chromosomes 2 and 4, respectively, in mitotic metaphases. In addition, the subtelomeric signal on 5S was useful for the recognition of chromosome 5. The somatic chromosomes 7 and 8 were also difficult to distinguish in lines without K7S, and then, the presence of the subtelomeric sequence at 8L was an important marker enabling the identification of this chromosome. It is interesting to note that in the analyses of chromosome 7 aberrations that were induced in callus cultures derived from JD 1-3 genotypes (Fluminhan et al., 1996; Gardingo et al., 2013), the chromosome 7 was easily distinguished in C-banded metaphases because it possessed knobs on 7S in all of the lines of the JD 1-3 family. Additionally, chromosomes 9 and 10 are difficult to distinguish based on of their morphology in lines in which chromosome 9 does not have the large terminal knob on the short arm, but in FISH preparations, it can be identified by the presence of the small signal of the 180-bp repeat on the short arm.

CentC hybridization signals were very bright and did not discriminate specific chromosomes. We interpret that this finding might be due to the detection technique using three antibodies, which would mask possible differences in the present work. The copy number of CentC units is variable in non-homologous maize chromosomes and among varieties and this variability has been shown with direct fluorophore-label, which would be more sensitive to detect differences among chromosomes and varieties as reported (Kato et al., 2004; Birchler and Han, 2009).

The presence of the subtelomeric 4-12-1 signal on 1S, 2S, 4SL, 5S and 8L in all of the JD lines and in KYS provided evidence that the copy numbers of the repeat unit in these 4-12-1 arrays allow for their detection at the resolution of the mitotic chromosomes, and that they may probably be detected in these positions in various maize varieties. They have been observed in maize lines, but varying among materials (Kato et al., 2004; Albert et al., 2010), including the KYS, in which we observed the subtelomeric signal at 5S and 5L.

Also, the observation of small knob signals on 1S, 6S, and 9S in the JD lines provided additional evidence that at these positions, there would be a detectable copy number of the 180-bp sequence in the chromosomes of several maize varieties as shown in the literature (Kato et al., 2004; Albert et al., 2010). Interestingly, these small heterochromatic sites coincide with those previously reported as “enlarged chromomeres,” observed in carmine-stained pachytene chromosomes (Dempsey, 1994; Neuffer et al., 1997). Signals for the 180-bp knob repeat and TR-1 element have been detected on the chromosome 6 short arm in all maize lines so far investigated (Ananiev et al., 1998a; Kato et al., 2004; Albert et al., 2010). Moreover, small knob signals have been found in several maize chromosome locations. Using a FISH procedure with increased sensitivity (involving increase in the exposure time) 180-bp repeats were detected near the ends of almost every somatic chromosome arm and on interstitial sites on pachytene chromosomes, which do not correspond with visible knobs (Lamb et al., 2007b). However, cytologically visible knobs in maize, which are sites with high copy numbers of 180-bp repeats, have been located at specific positions. In an extensive survey of maize races from North, Central and South America, 23 possible knob positions distributed among maize chromosomes were recognized, most of which were located at subterminal regions, suggesting the occurrence of strong selection pressure at these locations (McClintock et al., 1981).

The origin of knob polymorphism in maize and its wild teosinte progenitors, including number and size, has been discussed in several reports. Buckler et al. (1999) proposed that meiotic drive was responsible for the evolution of maize knobs. This meiotic event is a mechanism by which small regions of the genome are preferentially transmitted to the progeny. In maize, meiotic drive is due to an uncommon form of chromosome 10, or abnormal chromosome 10 (Ab 10), which causes the knobbed chromosomes to preferentially segregate during female meiosis because in the presence of this chromosome, the knobs of the other chromosomes are converted into neocentromeres (see Kikudome, 1959; Dawe and Hiatt, 2004). Other factors, like environment and transposition, may also have played a role in the evolution of knobs (Buckler et al., 1999). In their survey of maize races, McClintock et al. (1981) observed characteristic chromosomal patterns that suggested the existence of karyotype groups in some geographic regions. For example, in the so-called “Andean complex,” most of the examined races collections that were made at highlands in South America, had the same or nearly the same karyotype characteristics, including small knobs at 6L3 and 7L (McClintock, 1978). In our study, the knob composition of the JD 1-3 and JD 4-4 lines, derived from a S2 progeny, which was segregating for knobs at 3L, 5L, 7S, and 9S, is representative of the karyotype variability in sister homozygous inbred lines obtained after selfing cycles. Additionally, the relative sizes of the knobs were maintained invariable throughout these cycles. For example, K7L was always larger than the band that was observed at 8L, and K9S, when present, was a very large knob (**Figures 3** and **4**). All of the knobs scored in JD lines were consistent with those that were reported in collections of Cateto and Cuba races examined by McClintock et al. (1981), which were components of the original JD population. The knob on 7S is not frequently observed, but it was also described in some accessions of Cateto.

Knobs have been located on cytogenetical maps based on pachytene analyses (Longley, 1939). Recently they have been placed on the genetic map (Lawrence et al., 2006), and mapped relative to the maize reference genome assembly (Ghaffari et al., 2013). The authors used FISH to map visible knobs in recombinant inbred lines, and three knobs from the B73 inbred were accurately placed on the B73 reference genome. These data demonstrated that knobs lie in gene-dense regions, generally high recombination areas. Using their mapping data in combination with the NAM metapopulation, the authors compared recombination frequencies in the presence and absence of knobs, and revealed that knobs in heterozygous condition can reduce local recombination. Therefore, the knowledge of knob constitution of inbreds can be quite useful in breeding programs. Interestingly, during the development of inbred lines from the JD population, in S6 and even S9 progenies heterozygous plants for at least one knob position were detected in some progenies (Decico, 1991).

Knobs can also vary in their molecular structure among chromosomes and varieties. They are composed primarily of 180-bp repeats and TR-1 elements, and individual knobs can either be composed exclusively of 180-bp repeats or TR-1 elements or contain a mixture of both (Ananiev et al., 1998a; Kato et al., 2004; Albert et al., 2010; Ghaffari et al., 2013). TR-1 elements have been found in knobs on 2L, 4L, and 6S in most lines that have been investigated, but they were also detected on 6L, 8L, 9S, and 10L2 in some lines (Kato et al., 2004; Albert et al., 2010; Ghaffari et al., 2013; Kanizay et al., 2013). Our observation of large 180-bp signals corresponding with positions and sizes of knobs visible at pachytene stage and in C-banded somatic metaphases suggests that these knobs are composed primarily of 180-bp repeats. In the materials studied here, knobs on 2L, 4L, and 10L were not observed, but further investigation is necessary to assess whether TR-1 elements are present on 6S, 6L2, 6L3, 8L1, 8L2, and 9S.

### BEHAVIOR OF THE CHROMOSOMES 2 AND 4 AT PACHYTENE STAGE IN THE 441311 × KYS HYBRID AND INFERENCES ON CHROMOSOME EVOLUTION

The arm ratios estimated for chromosomes 2 and 4 at pachytene stage gave evidence of differences in the centromere position of these chromosomes between the JD and KYS lines (**Table 3**). In the present study, based on the arm ratios, we found that the centromere position of the chromosome 2 in the 441311 × KYS hybrid was similar to that observed in the 441311 line, while the arm ratio of chromosome 4 was intermediate between values estimated for JD and KYS lines. In addition, both homologues of pair 2 and of pair 4 were completely synapsed. The only exception was observed in a meiotic cell showing a loop in the chromosome pair 2, suggesting a failure of pairing (**Figure 5B**). FISH using satellite DNA probes revealed complete synapsis at homologous marker sites, i.e., CentC and 5S rDNA in chromosome 2, and Cent4 and subtelomeric 4-12-1 in chromosome 4. It is beyond the scope of our study to discuss the mechanism involved in the pairing of homologous chromosomes differing in centromere positions, which was observed in the 441311 × KYS hybrid, but this finding raises some questions that may be considered in further investigations.

Alterations in the arm lengths of a chromosome can occur due to the presence of a pericentric inversion or a deficiency or duplication of a chromosomal segment. In inversion heterozygotes a loop can be detected at pachynema, when a standard chromosome pairs with the homologue containing the inversion, allowing for homologous pairing in the inverted region. In deficiency and duplication heterozygotes, a loop formed by an unpaired segment can be observed. In the 441311 × KYS hybrid, loops were not detected in pairs 2 and 4 and this suggests that the homologous chromosomes could pair through a differential degree of chromatin packaging between homologous arms differing in size. Therefore, the loop observed in the chromosome 2 in one cell (**Figure 5B**) would be an exceptional event of pairing failure. The axial contraction along the fibers during meiotic mid-prophase has been shown to be uniform, using BAC FISH mapping of selected loci on maize chromosome addition lines of oats (Figueroa and Bass, 2012). In their study, the authors found that the relative loci positions along pachytene chromosomes did not change as a function of total arm length at early and late pachynema. However, they observed considerable variation between the relative arm positions of loci when comparing the cytogenetic FISH map to the B73 genomic physical map. As mentioned by the authors, this could occur in some cases in which the cytogenetic FISH map and genomic physical map are from different genotypes. Differences in genomic content among maize lines is well known, for example, the genome size of the Mo17 and B73 lines are estimated to differ by 0.13 pg (see Figueroa and Bass, 2012), and highly significant variation in 4C DNA content in maize varieties, ranging from 9.84 to 13.49 pg, has been reported (Laurie and Bennett, 1985). According to Figueroa and Bass (2012) variation in relative map positions could result from genotype-specific variation in DNA packaging along the pachytene chromosome axis of individual chromosome arms. Alternatively, the authors argued that genome sizes could be similar, but that repetitive DNA sequences may have accumulated in different regions of the chromosome arms. Satellite DNA and TEs were identified in inbred line B73, and it was estimated that ∼85% of the B73 RefGen_v1 were comprised of TEs, of which 75% belonged to LTR retrotransposon families (Schnable et al., 2009). In addition, the distribution of some of the retrotransposon families on the chromosomes of maize lines were shown to be non-random, with distinct patterns revealed by FISH (Lamb et al., 2007a ).

From this scenario, we could infer that the KYS and JD lines have different contents of repetitive DNA along the arms of the knobless chromosomes 2 and 4, resulting in differences in relative arm lengths. It is interesting to note that two different arm ratio values have been reported for chromosomes 2 and 4 (Neuffer et al., 1997), but with values in chromosome 4 being higher than in chromosome 2. The complete synapsis that we observed at pachytene stage may involve non-homologous pairing at some chromosome regions; however, the homologous pairing at the satellite DNA regions was remarkable. Synapsis of non-homologous parts of chromosomes in pachynema has been detected in maize. The first report of this behavior (McClintock, 1933) showed several instances of non-homologous synapsis in heterozygotes for deficiencies and inversions, and within the univalent in monosomic plants and in parts of the homologues in trisomic plants. In addition, other cases of non-homologous pairing in maize have been reported, such as that which was visualized using FISH in a heterozygote for a hemicentric inversion in chromosome 8, involving pericentromeric heterochromatin (Lamb et al., 2007a).

In conclusion, the results of our study highlight problems to be investigated concerning meiotic chromosome synapsis and levels of chromatin contraction along chromosomes with different distributions of repetitive DNA, besides contributing for the knowledge of global maize chromosome variability.

## AUTHOR CONTRIBUTIONS

Mateus Mondin and Janay A. Santos-Serejo performed FISH experiments and karyotype analysis; Mônica R. Bertão performed cytological work and karyotype analysis; Prianda Laborda isolated the CentC probe; Janay A. Santos-Serejo and Daniel Pizzaia performed pachytene analysis; Margarida L. R. Aguiar-Perecin performed research work and wrote the paper.

## Conflict of Interest Statement

The authors declare that the research was conducted in the absence of any commercial or financial relationships that could be construed as a potential conflict of interest.

## Supplementary material

Figure S1 **Origin and C-band (knob) composition of the S6 inbred line families JD 1-3 and JD 4-4 derived from a S2 progenitor, which segregated for knobs on 3L, 5L, 7S, and 9S and was homozygous for knobs on 6L, 7L, and 8L**.

Figure S2 **CentC probe consisting of two CentC consensus repeats (Ananiev et al., 1998b) of 139-bp and 154-bp respectively (red)**.

## References

[B1] Aguiar-Perecin M. L. R. Fluminhan A. Santos-Serejo J. A. Gardingo J. R. Bertão M. R. Decico M. J. U. (2000). Heterochromatin of maize chromosomes: structure and genetic effects. *Genet. Mol. Biol.* 23 1015–1019 10.1590/S1415-47572000000400047

[B2] Aguiar-Perecin M. L. R. Vosa C. G. (1985). C-banding in maize II. Identification of somatic chromosomes. *Heredity* 54 37–42 10.1038/hyd.1985.6

[B3] Albert P. S. Gao Z. Danilova T. V. Birchler J. A. (2010). Diversity of chromosomal karyotypes in maize and its relatives. *Cytogenet. Genome. Res.* 129 6–16 10.1159/000314342 20551613

[B4] Ananiev E. V. Phillips R. L. Rines H. W. (1998a). A knob-associated tandem repeat in maize capable of forming fold-back DNA segments: are chromosome knobs megatransposons? *Proc. Natl. Acad. Sci. U.S.A.* 95 10785–10790 10.1073/pnas.95.18.10785 9724782 PMC27973

[B5] Ananiev E. V. Phillips R. L. Rines H. W. (1998b). Chromosome-specific molecular organization of maize (*Zea mays* L.) centromeric regions. *Proc. Natl. Acad. Sci. U.S.A.* 95 13073–13078 10.1073/pnas.95.22.13073 9789043 PMC23713

[B6] Anderson L. K. Doyle G. G. Brigham B. Carter J. Hooker K. D. Lai A. (2003). High-resolution crossover maps for each bivalent of *Zea mays* using recombination nodules. *Genetics* 165 849–865 14573493 10.1093/genetics/165.2.849 PMC1462767

[B7] Anderson L. K. Salameh N. Bass H. W. Harper L. C. Cande W. Z. Weber G. (2004). Integrating genetic linkage maps with pachytene chromosome structure in maize. *Genetics* 166 1923–1933 10.1534/genetics.166.4.1923 15126409 PMC1470829

[B8] Bertão M. R. Aguiar-Perecin M. L. R. (2002). Maize somatic chromosome preparation: pretreatments and genotypes for obtention of high index of metaphases accumulation. *Caryologia* 55 115–119 10.1080/00087114.2002.10589266

[B9] Birchler J. A. Han F. (2009). Maize centromeres: structure, function, epigenetics. *Annu. Rev. Genet.* 43 287–303 10.1146/annurev-genet-102108-134834 19689211

[B10] Buckler E. S. Phelps-Durr T. L. Buckler C. S. K. Dawe R. K. Doebley J. F. Holtsford T. P. (1999). Meiotic drive of chromosomal knobs reshaped the maize genome. *Genetics* 153 415–426 10471723 10.1093/genetics/153.1.415 PMC1460728

[B11] Carlson W. R. (1988). “The cytogenetics of corn,” in *Corn and Corn Improvement* eds Sprague G. F. Dudley J. W. (Madison, WI: American Society of Agronomy) 259–343

[B12] Chen C. C. Chen C. M. Hsu F. C. Wang C. J. Yang J. T. Kao Y. Y. (2000). The pachytene chromosomes of maize as revealed by fluorescence in situ hybridization with repetitive DNA sequences. *Theor. Appl. Genet.* 101 30–36 10.1007/s001220051445

[B13] Coe E. H. (1994). “A-A translocations: breakpoints and stocks,” in *The Maize Handbook* eds Frelling M. Walbot V. (New York, NY: Springer) 364–376

[B14] Creighton H. B. McClintock B. (1931). A correlation of cytological and genetic crossing-over in *Zea mays*. *Proc. Natl. Acad. Sci. U.S.A.* 17 492–497 10.1073/pnas.17.8.492 16587654 PMC1076098

[B15] Danilova T. V. Birchler J. A. (2008). Integrated cytogenetic map of mitotic metaphase chromosome 9 of maize: resolution, sensitivity, and banding paint development. *Chromosoma* 117 345–356 10.1007/s00412-008-0151-y 18317793

[B16] Dawe R. K. Hiatt E. N. (2004). Plant neocentromeres: fast, focused and driven. *Chromosome Res.* 12 655–669 10.1023/B:CHRO.0000036607.74671.DB 15289670

[B17] Decico M. J. U. (1991). *Análise da segregação de knobs em progênies F2 e de retrocruzamento derivadas de uma variedade de milho*. MS thesis, Luiz de Queiroz College of Agriculture, University of São Paulo, Piracicaba.

[B18] Dempsey E. (1994). “Traditional analysis of maize pachytene chromosomes,” in *The Maize Handbook* eds Freeling M. Walbot V. (New York, NY: Springer-Verlag) 432–441

[B19] Faraway J. J. (2004). *Linear Models with R.* London: Chapman & Hall/CRC

[B20] Figueroa D. M. Bass H. W. (2012). Development of pachytene FISH maps for six maize chromosomes and their integration with other maize maps for insights into genome structure variation. *Chromosome Res.* 20 363–380 10.1007/s10577-012-9281-4 22588802 PMC3391363

[B21] Fluminhan A. Aguiar-Perecin M. L. R. (1998). Embryogenic response and mitotic instability in callus cultures derived from maize inbred lines differing in heterochromatic knob content of chromosomes. *Ann. Bot.* 82 569–576 10.1006/anbo.1998.0710

[B22] Fluminhan A. Aguiar-Perecin M. L. R. Santos J. A. (1996). Evidence for heterochromatin involvement in chromosome breakage in maize callus culture. *Ann. Bot.* 78 73–81 10.1006/anbo.1996.0098

[B23] Gardingo J. Santos-Serejo J. A. Aguiar-Perecin M. L. R. (2013). Amplification of heterochromatic knob size in callus culture by unequal sister chromatid exchange. *Maize Genet. Coop. Newslett.* 86 26

[B24] Ghaffari R. Cannon E. K. S. Kanizay L. B. Lawrence C. J. Dawe R. K. (2013). Maize chromosomal knobs are located in gene-dense areas and suppress local recombination. *Chromosoma* 122 67–75 10.1007/s00412-012-0391-8 23223973 PMC3608884

[B25] Heslop-Harrison J. S. Schwarzacher T. (2011). Organization of the plant genome in chromosomes. *Plant J.* 66 18–33 10.1111/j.1365-313X.2011.04544.x 21443620

[B26] Hoisington D. Khairallah M. Gonzáez-de-Léon D. (1994). *Laboratory Protocols: CIMMYT Applied Molecular Genetics Laboratory* 2nd Edn. Texcoco: CIMMYT

[B27] Jin W. Melo J. R. Nagaki K. Talbert P. B. Henikoff S. Dawe R. K. (2004). Maize centromeres: organization and functional adaptation in the genetic background of oat. *Plant Cell* 16 571–581 10.1105/tpc.018937 14973167 PMC385273

[B28] Kanizay L. B. Albert P. S. Birchler J. A. Dawe R. K. (2013). Intragenomic conflict between the two major knob repeats of maize. *Genetics* 194 81–19 10.1534/genetics.112.148882 23457233 PMC3632483

[B29] Kato A. Lamb J. C. Birchler J. A. (2004). Chromosome painting using repetitive DNA sequences as probes for somatic chromosome identification in maize. *Proc. Natl. Acad. Sci. U.S.A.* 101 13554–13559 10.1073/pnas.0403659101 15342909 PMC518793

[B30] Kikudome G. Y. (1959). Studies on the phenomenon of preferential segregation in maize. *Genetics* 44 815–831 17247860 10.1093/genetics/44.5.815 PMC1209984

[B31] Lamb J. C. Birchler J. A. (2006). Retroelement genome painting: cytological visualization of retroelement expansions in the genera *Zea* and *Tripsacum*. *Genetics* 173 1007–1021 10.1534/genetics.105.053165 16582446 PMC1526525

[B32] Lamb J. C. Meyer J. M. Birchler J. A. (2007a). A hemicentric inversion in the maize line knobless Tama flint created two sites of centromeric elements and moved the kinetochore-forming region. *Chromosoma* 116 237–247 10.1007/s00412-007-0096-6 17256108

[B33] Lamb J. C. Meyer J. M. Corcoran B. Kato A. Han F. Birchler J. A. (2007b). Distinct chromosomal distributions of highly repetitive sequences in maize. *Chromosome Res.* 15 33–49 10.1007/s10577-006-1102-1 17295125

[B34] Laurie D. A. Bennett M. D. (1985). Nuclear DNA content in the genera *Zea* and *Sorghum*: intergeneric, interspecific and intraspecific variation. *Heredity* 55 307–313 10.1038/hdy.1985.112

[B35] Lawrence C. J. Seigfried T. E. Bass H. W. Anderson L. K. (2006). Predicting chromosomal locations of genetically mapped loci in maize using the Morgan2McClintock Translator. *Genetics* 172 2007–2009 10.1534/genetics.105.054155 16387866 PMC1456272

[B36] Longley A. E. (1939). Knob position on corn chromosomes. *J. Agric. Res.* 59 475–490

[B37] Mascia P. N. Rubenstein I. Phillips R. L. Wang A. S. Xiang L. Z. (1981). Localization of the 5S rRNA genes and evidence for diversity in the 5S rDNA region of maize. *Gene* 15 7–20 10.1016/0378-1119(81)90099-8 6170540

[B38] McClintock B. (1930). A cytological demonstration of the location of an interchange between two non-homologous chromosomes of *Zea mays*. *Proc. Natl. Acad. Sci. U.S.A.* 16 791–796 10.1073/pnas.16.12.791 16577311 PMC544331

[B39] McClintock B. (1933). The association of non-homologous parts of chromosomes in mid-prophase of meiosis in *Zea mays*. *Z. Zellforsch. Mikrosk. Anat.* 19 191–237 10.1007/BF02462870

[B40] McClintock B. (1950). The origin and behavior of *mutable* loci in maize. *Proc. Natl. Acad. Sci. U.S.A.* 36 344–355 10.1073/pnas.36.6.344 15430309 PMC1063197

[B41] McClintock B. (1978). “Significance of chromosome constitutions in tracing the origin and migration of races of maize in the Americas,” in *Maize Breeding and Genetics* ed. Walden D. B. (New York, NY: John Wiley & Sons) 159–184

[B42] McClintock B. Kato T. A. Blumenschein A. (1981). *Chromosome Constitution of Races of Maize.* Chapingo: Colegio de Postgraduados

[B43] Mondin M. Santos-Serejo J. A. Aguiar-Perecin M. L. R. (2007). Karyotype characterization of *Crotalaria juncea* (L.) by chromosome banding and physical mapping of 18S-5.8S-26S and 5S rRNA gene sites. *Genet. Mol. Biol.* 30 65–72 10.1590/S1415-47572007000100013

[B44] Nagaki K. Song J. Stupar S. M. Parokonny A. S. Yuan Q. Ouyang S. (2003). Molecular and cytological analyses of large tracks of centromeric DNA reveal the structure and evolutionary dynamics of maize centromeres. *Genetics* 163 759–770 12618412 10.1093/genetics/163.2.759 PMC1462457

[B45] Neuffer M. G. Coe E. H. Wessler S. R. (1997). *Mutants of Maize.* Plainview, NY: Cold Spring Harbor Laboratory Press

[B46] Page B. T. Wanous M. K. Birchler J. (2001). Characterization of a maize chromosome 4 centromeric sequence: evidence for an evolutionary relationship with the B chromosome centromere. *Genetics* 159 291–302 11560905 10.1093/genetics/159.1.291 PMC1461786

[B47] Peacock W. J. Dennis E. S. Rhoades M. M. Pryor A. J. (1981). Highly repeated DNA sequence limited to knob heterochromatin. *Proc. Natl. Acad. Sci. U.S.A.* 78 4490–4494 10.1073/pnas.78.7.4490 16593063 PMC319817

[B48] Rayburn A. L. Price H. J. Smith J. D. Gold J. R. (1985). C-band heterochromatin and DNA content in *Zea mays*. *Am. J. Bot.* 72 1610–1617 10.2307/2443312

[B49] Rhoades M. M. (1950). Meiosis in maize. *J. Hered.* 41 58–67 10.1093/oxfordjournals.jhered.a106089 15422115

[B50] Schnable P. S. Ware D. Fulton R. S. Stein J. C. Wei F. Pasternak S. (2009). The B73 maize genome: complexity, diversity, and dynamics. *Science* 326 1112–1115 10.1126/science.1178534 19965430

[B51] Zhong C. X. Marshall J. B. Topp C. Mroczek R. Kato A. Nagaki K. (2002). Centromeric retroelements and satellites interact with maize kinetochore protein CENH3. *Plant Cell* 14 2825–2836 10.1105/tpc.006106 12417704 PMC152730

