# Supplementary figures and images for: Karyotype variability in tropical maize sister inbred lines and hybrids compared with KYS standard line
Source: Front Plant Sci. 2014 Oct 13;5:544. doi: 10.3389/fpls.2014.00544 (PMC4195276; doi:10.3389/fpls.2014.00544)

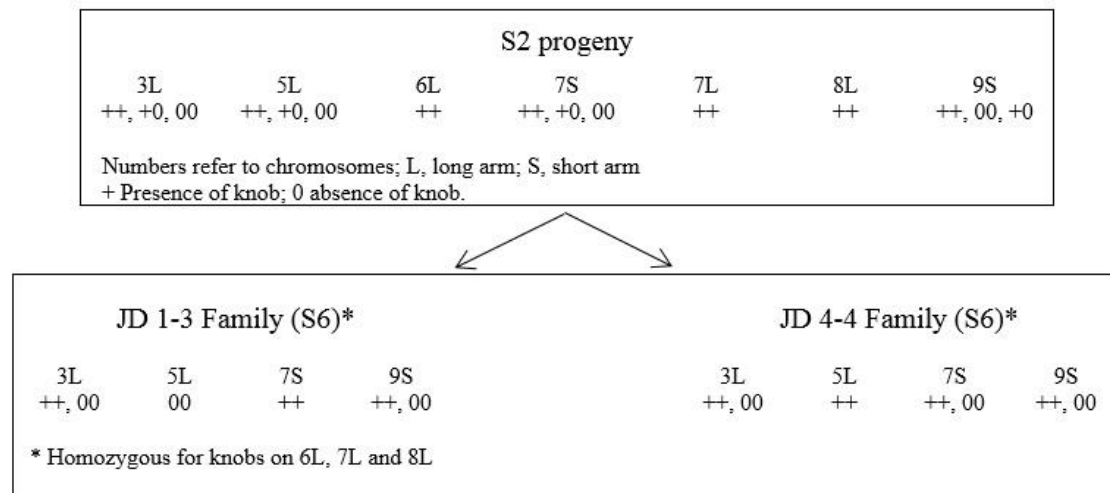

Supplement: Figure S1 — Origin and C-band (knob) composition of the S6 inbred line families JD 1-3 and JD 4-4 derived from a S2 progenitor, which segregated for knobs on 3L, 5L, 7S, and 9S and was homozygous for knobs on 6L, 7L, and 8L. [file Data_Sheet_1.PDF]
