# Supplementary material for: Karyotype variability in tropical maize sister inbred lines and hybrids compared with KYS standard line
Source: Front Plant Sci. 2014 Oct 13;5:544. doi: 10.3389/fpls.2014.00544 (PMC4195276; doi:10.3389/fpls.2014.00544)
Supplement: Figure S2 — CentC probe consisting of two CentC consensus repeats (Ananiev et al., 1998b) of 139-bp and 154-bp respectively (red). [file Data_Sheet_2.DOCX]

5**’ACTCGTGCTTTTTATGCACCCTCGACAACCGTTTTCAGAATGGGTGACGTGCGGAAACGAAATTGCGCGAAAACAACCCAAACATGAGTTTAGTACCTAAAGTAGTGGATTGGGCATGTTCGTTGCGAAAAACGAAGAAGTTGTTCCGGTGGCAAAAACTCGTGCTTTGTATGCACCCGATACCCGTTTTCGGAATTGGTGACATGCGCCAACGAAATTGCGTGAAACCACACCAAACATGAGTTTTGTACCTAAAGTAGTGGATTGGGCATGTTCGTTGTGAAAAACGAAGAAAT3’**
